# New-onset psychosis following COVID-19 vaccination: a systematic review

**DOI:** 10.3389/fpsyt.2024.1360338

**Published:** 2024-04-12

**Authors:** Marija Lazareva, Lubova Renemane, Jelena Vrublevska, Elmars Rancans

**Affiliations:** ^1^ Department of Psychiatry and Narcology, Riga Stradins University, Riga, Latvia; ^2^ Residency in Psychiatry Program, University of Latvia, Riga, Latvia

**Keywords:** SARS-CoV-2, COVID-19, vaccination, new-onset psychosis, adverse effects

## Abstract

**Background:**

The emergence of a new coronavirus strain caused the COVID-19 pandemic. While vaccines effectively control the infection, it’s important to acknowledge the potential for side effects, including rare cases like psychosis, which may increase with the rising number of vaccinations.

**Objectives:**

Our systematic review aimed to examine cases of new-onset psychosis following COVID-19 vaccination.

**Methods:**

We conducted a systematic review of case reports and case series on new-onset psychosis following COVID-19 vaccination from December 1st, 2019, to November 21st, 2023, using PubMed, MEDLINE, ClinicalKey, and ScienceDirect. Data extraction covered study and participant characteristics, comorbidities, COVID-19 vaccine details, and clinical features. The Joanna Briggs Institute quality assessment tools were employed for included studies, revealing no significant publication bias.

**Results:**

A total of 21 articles described 24 cases of new-onset psychotic symptoms following COVID-19 vaccination. Of these cases, 54.2% were female, with a mean age of 33.71 ± 12.02 years. Psychiatric events were potentially induced by the mRNA BNT162b2 vaccine in 33.3% of cases, and psychotic symptoms appeared in 25% following the viral vector ChAdOx1 nCoV-19 vaccine. The mean onset time was 5.75 ± 8.14 days, mostly reported after the first or second dose. The duration of psychotic symptoms ranged between 1 and 2 months with a mean of 52.48 ± 60.07 days. Blood test abnormalities were noted in 50% of cases, mainly mild to moderate leukocytosis and elevated C-reactive protein. Magnetic resonance imaging results were abnormal in 20.8%, often showing fluid-attenuated inversion recovery hyperintensity in the white matter. Treatment included atypical antipsychotics in 83.3% of cases, typical antipsychotics in 37.5%, benzodiazepines in 50%, 20.8% received steroids, and 25% were prescribed antiepileptic medications. Overall, 50% of patients achieved full recovery.

**Conclusion:**

Studies on psychiatric side effects post-COVID-19 vaccination are limited, and making conclusions on vaccine advantages or disadvantages is challenging. Vaccination is generally safe, but data suggest a potential link between young age, mRNA, and viral vector vaccines with new-onset psychosis within 7 days post-vaccination. Collecting data on vaccine-related psychiatric effects is crucial for prevention, and an algorithm for monitoring and treating mental health reactions post-vaccination is necessary for comprehensive management.

**Systematic review registration:**

https://www.crd.york.ac.uk/PROSPERO, identifier CRD42023446270.

## 1 Introduction

The end of 2019 was marked by an event that radically changed the lives of all humanity. The appearance of a new strain of coronavirus, subsequently termed severe acute respiratory syndrome coronavirus 2 (SARS-CoV-2), led to a pandemic of the disease called COVID-19. According to a World Health Organization (WHO) report, as of October 2023, there have been 771,191,203 confirmed cases of COVID-19 globally, including 6,961,014 deaths (1).

As a result, there was a critical need to focus on vaccinating the population against SARS-CoV-2 infection, reducing the risk of severe disease and mortality. The first vaccine, the Pfizer-BioNTech COVID-19 Vaccine COMIRNATY (mRNA BNT162b2 vaccine), was approved by the WHO in December 2020. The United States Food and Drug Administration (FDA) granted emergency use authorization for COVID-19 vaccines and full approval for the Pfizer vaccine to control the pandemic. The WHO has also recommended other vaccines for COVID-19 produced by numerous pharmaceutical companies (2). Vaccines against COVID-19 differ in composition and mechanism of action, which may be relevant for their safety and efficacy (3). As of October 2023, the WHO reported a total of 13,516,185,809 vaccine doses administered (1).

Despite the widespread use and high effectiveness of vaccines in controlling COVID-19 infection, it is crucial to consider the possibility of side effects. To date, no vaccine can be claimed to be completely free of adverse events, but fortunately, the majority of them are either preventable or treatable (4). Most early side effects, such as fever, pain, myalgias, headaches, and local or injection side effects, are related to the immune response and are considered common (5). However, several studies have demonstrated cardiac, gastrointestinal, neurological, and psychiatric side effects associated with COVID-19 vaccines (6–10). The recent review describes 14 cases of altered mental states, psychosis, affective, and functional neurological disorders as psychiatric and neuropsychiatric adverse reactions to mRNA or vector-based COVID-19 vaccines (11). As the number of vaccinated people increases, so does the number of reported cases of rare vaccine-related side effects, such as psychosis. Therefore, we conducted a systematic review to examine cases of new-onset psychosis following COVID-19 vaccination with all types of vaccines. It is worth noting that our study is the first of its kind, and, in prospect, will help expand the understanding of rare and clinically significant side effects of COVID-19 vaccines.

## 2 Methods

### 2.1 Study design

The present study systematically reviewed the case reports and case series of new-onset psychosis associated with COVID-19 vaccination. The protocol of this systematic review was registered in the PROSPERO database with ID number: CRD42023446270. A systematic search was carried out following the Preferred Reporting Items for Systematic Reviews and Meta-analyses (PRISMA) (12).

### 2.2 Search strategy

For the review, the following databases for relevant English language literature were searched: PubMed, MEDLINE, ClinicalKey, and ScienceDirect. Studies were restricted to those published from December 1st, 2019 (the beginning of the COVID-19 outbreak) to November 21st, 2023. A systematic search was carried out to select studies that corresponded to the inclusion and exclusion criteria using keywords, generic filters, and MeSH terms in the referred databases, following the PRISMA guidelines. The electronic database search was supplemented by a manual search of the reference lists of included articles and Google Scholar.

The following keywords for search strategy were used: COVID-19 vaccine* OR SARS-CoV-2 vaccine* OR coronavirus vaccine* OR mRNA vaccine* OR BNT162b2 vaccine* OR viral vector vaccine* OR ChAdOx1-S/nCoV-19 vaccine* OR Ad26.COV2.S vaccine* OR Whole-virion inactivated vaccine* OR BBV152 vaccine* AND psychosis OR psychotic disorders OR psychiatric disorders OR neuropsychiatric OR hallucinations OR mania OR delusions OR schizophrenia OR mental disorders OR psychomotor disorder OR confusion OR delirium OR agitation.

The following inclusion criteria were used: all studies conducted in primary, secondary, and tertiary care settings, with no restrictions on the location of the healthcare setting; eligible studies must include all patients who received the COVID-19 vaccine and exhibited psychotic symptoms according to the Diagnostic and Statistical Manual of Mental Disorders V or the International Classification of Diseases 10/11, with no restrictions on gender, age, or race.

The exclusion criteria were as follows: reviews, clinical trials, letters to the editor, editorials, interviews, newspaper articles, comments, studies without sufficient data, duplicate sources, as well as studies with only a published abstract. Additionally, studies that included patients with diagnosed psychiatric disorders/psychotic symptoms before COVID-19 vaccination or patients with no history of COVID-19 vaccination were excluded.

### 2.3 Data extraction and analysis

The study selection process involved two stages: initially, two independent reviewers (M.L., L.R.) screened study titles, and then the full texts of the selected articles were independently assessed for eligibility. Articles failing to meet the inclusion criteria or those that were unavailable were excluded by the reviewers. The screening results and data extraction underwent verification by two additional reviewers (J.V., E.R.), who were not part of the initial screening and extraction process. Any disagreements were resolved through discussion until a consensus was reached.

Reviewers extracted data from the primary studies in the following domains: 1) study characteristics (i.e., principal author, country, year of publication, study design), 2) characteristics of participants (i.e., age and gender), 3) comorbidities (i.e., history of somatic disorders), 4) characteristics of the COVID-19 vaccine (i.e., type and name, dose of vaccine after which psychotic symptoms appeared), 5) clinical characteristics (time to psychotic symptoms from vaccine administration, morbidity and/or diagnosis, laboratory findings and/or imaging, duration of psychotic episode, medications used, and outcome).

Descriptive analysis of the available information was performed using descriptive statistics to report demographics and clinical characteristics. Authors reported the onset and duration of symptoms as a range due to inconsistent and approximate reporting of this information across studies.

### 2.4 Quality assessment

The Joanna Briggs Institute (JBI) quality assessment tools were used for the case reports and case series included in our study (13, 14). The quality of the included studies was assessed by two authors, and conflicts were resolved by consensus. For case reports, JBI domains included eight questions, and for assessing case series, ten questions were used. Quality assessment was carried out in the following categories: 75-100% low risk; 50-74% moderate risk; <50% high risk.

## 3 Results

### 3.1 Study characteristics

**Figure 1:**
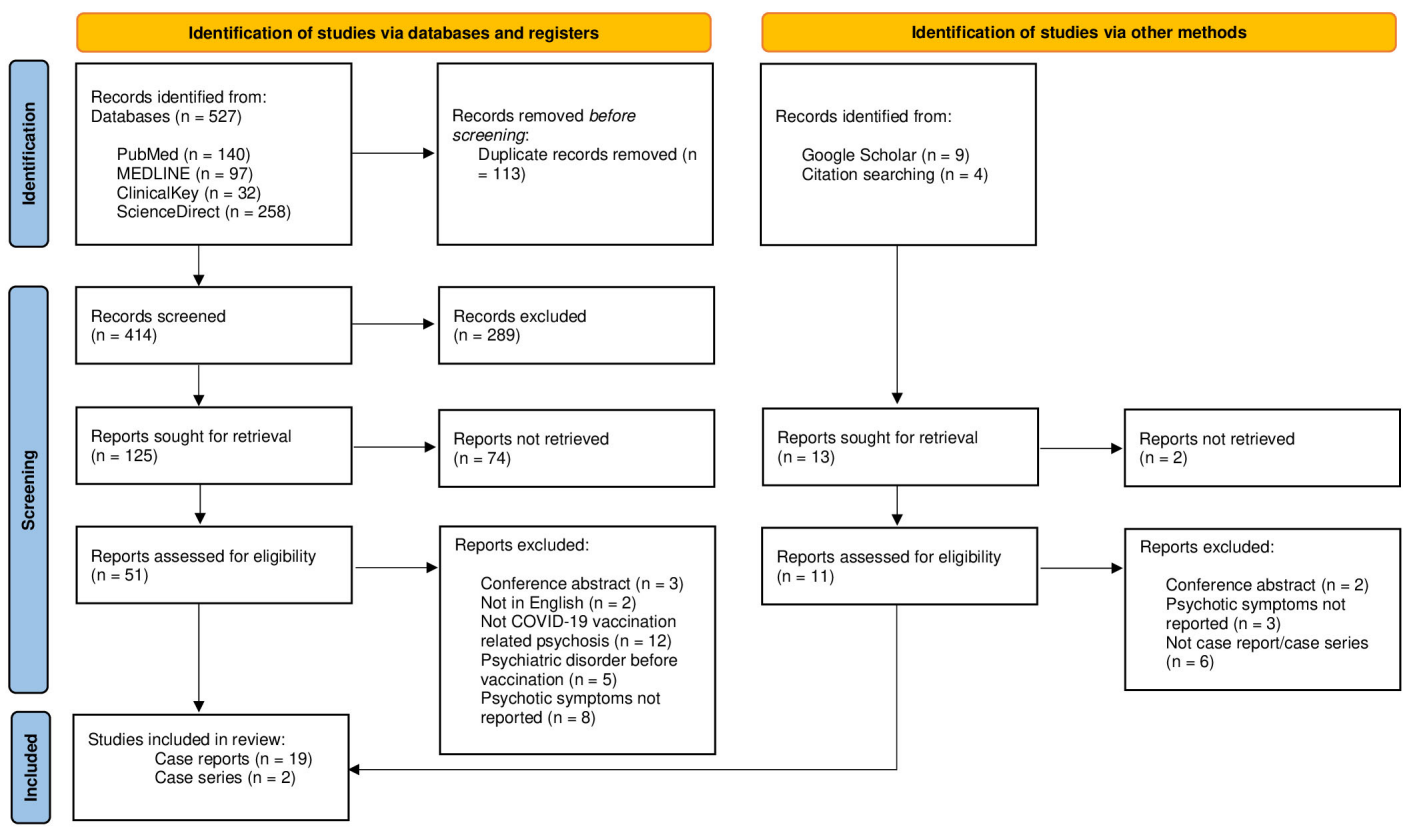
PRISMA flow diagram.

A total of 21 articles (10, 15–34) describing 24 cases of new-onset psychotic symptoms following COVID-19 vaccination were retrieved from PubMed, MEDLINE, ClinicalKey, ScienceDirect, and Google Scholar (see **Figure 1**). All studies were in English, and the largest number of cases were reported in the United States of America (12%) and India (20%) (see **Table 1**).

**Table 1:** Summary of studies of new-onset psychosis cases following COVID-19 vaccination.

Study reference	Age, sex, country	Type of vaccine, dose	Onset time	Psychiatric presentation	Diagnosis	Duration	Medical history, comorbidities	Pathological laboratory and radiological findings	Treatment and follow-up
Flannery et al. (15)	20/F/USA	mRNA BNT162b2 vaccine/1^st^ dose	1 week	Anxiety; insomnia; hypochondriacal delusions; auditory hallucinations; catatonia; aphasia.	Anti-NMDAR encephalitis	54 days	No	BT: mild leukocytosis, increased ALAT and ASAT. CSF: mild lymphocyte pleocytosis with 12–14 nucleated cells/mm3.	Olanzapine; Haloperidol; Lithium; Ziprasidone; Risperidone; IVIG; Methylprednisolone; Rituximab. Patient was discharged from the hospital with minor neurological deficits.
Reinfeld et al. (16)	31/M/USA	mRNA vaccine/1^st^ dose	NS	Auditory hallucinations; grandiose delusions; erotomaniac delusions; anxiety.	Unspecified psychosis	35 days	No	BT: moderate leukocytosis with left shift, ESR of 48 mm/h. CT: hyperintensities throughout the subcortical and periventricular white matter. MRI: FLAIR hyperintensity in the left peritrigonal white matter, with multiple nonspecific punctate hyperintensities throughout the subcortical and periventricular white matter and focus of susceptibility in the right lateral thalamus.	Risperidone. Patient was discharged with good insight about his symptoms. One week later he was taking medication, asymptomatic and back to work.
Yesilkaya et al. (33)	42/M/Turkey	mRNA BNT162b2 vaccine/1^st^ dose	1 day	Anxiety; increased psychomotor activity; agitation; irritability; insomnia; dysphoric mood; persecutory and referential delusions; loosening of associations.	Mania with psychotic symptoms	12 days	No	BT: elevated C-RP 4.2 mg/dL, WBC count was 8.8mg/dL	Olanzapine. 15 days after discharge, the patient declared that he was unable to remember the initiation of psychiatric symptoms.
	57/M/Turkey	mRNA BNT162b2 vaccine/2^nd^ dose	1 day	Increased psychomotor activity; anxiety; dysphoric mood; nihilistic delusions; irritability; insomnia; talking to self; suicidal attempt.	Mania with psychotic symptoms	8 days	No	Within normal limits.	Risperidone. Manic symptoms were improved.
Grover et al. (17)	18/F/India	Viral vector ChAdOx1 nCoV-19 vaccine/1^st^ dose	1 day	Irrelevant talk; bizarre behavior; irritability; anxiety; insomnia; delusions of persecution and reference; visual hallucinations; catatonia (mutism, staring, rigidity and negativism).	Unspecified psychosis	2 weeks	No	MRI: discrete tiny T2/FLAIR hyperintensities in deep white matter in bilateral fronto-parietal lobes.	Olanzapine; Lorazepam. 3 months after discharge the patient demonstrated good adherence and regular follow up. Patient refused to take the second dose of COVID-19 vaccine.
Lien et al. (18)	15/M/Taiwan	mRNA BNT162B2 vaccine/2^nd^ dose	2 days	Agitation; involuntary limb stretching; screaming; bizarre behavior; mannerism; auditory hallucinations; delusions.	Unspecified psychosis	2 months	No	BT: elevated CPK levels of 792U/L and CK-MB levels of 28 U/L.	Aripiprazole; Biperiden; Methylprednisolone. Hallucinations and bizarre behavior persisted for >1 month after discharge. The total course of steroid treatment was 3 weeks, and the patient’s psychiatric status gradually improved within the 2-month follow-up.
Takata et al. (19)	22/F/UK	Viral vector ChAdOx1 nCoV-19 vaccine/2^nd^ dose	Few days	Disorientation to time, person and place; agitation with a labile affect; auditory, visual and tactile hallucinations; hyper-religious delusions.	Limbic encephalitis	3 weeks	Non-syndromic retinitis pigmentosa	BT: Hb 126 g/L; WCC 6.6 × 109/L; CRP 1.1 mg/L; platelets 275 × 109/L; D-dimer 1,240 ng/mL. CSF: positive for IgG oligoclonal bands.	Paracetamol; Ceftriaxone; Acyclovir; Lorazepam; Haloperidol; Olanzapine; promethazine. One month after discharge the patient was functionally well with independent activities of daily living. Patient became quieter and more withdrawn, and still had residual psychotic symptoms of occasionally seeing green lights and praying more than before.
Roberts et al. (10)	51/M/UK	Viral vector ChAdOx1 nCoV-19 vaccine/1^st^ dose	10 days	Confusion and disorientation in time and place; speaking in short sentences, sometimes whispering or mumbling to being completely mute; disordered thinking; auditory hallucinations; bizarre and disinhibited behavior.	Unspecified psychosis	4 days	No	Within normal limits.	Lorazepam. One month post-discharge, patient reported feeling very well with no recurrence of symptoms.
Renemane et al. (20)	45/M/Latvia	mRNA vaccine/2^nd^ dose	Same day	Insomnia; unreasonable anxiety; tremor; disorientation in time; delusions of persecution; delusions of influence; thoughts insertion; delusional behavior; suicide attempt.	Acute and transient psychotic disorder	8 weeks	No	BT: WBC 12.8-10^3^µl	Metoprolol; Phenibut; Zolpidem tartrate; Haloperidol; Trihexyphenidyl; Diazepam; Quetiapine. The acute psychotic symptoms gradually resolved, and the patient was discharged from the hospital on the same medication regime with a good insight about his condition.
Alphonso et al. (21)	45/F/USA	mRNA-1273 vaccine/2^nd^ dose	1 month	Paranoia; auditory hallucinations; irrational fear; delusions of persecution; anxiety; agitation.	Unspecified psychosis	–	Diabetes mellitus II; hypothyroidism; hypertension	BT: low vitamin D level at 18.2 ng/mL	Quetiapine; Sertraline; Risperidone. The patient was referred to the neurology department for further imaging, autoimmune encephalitis panel, and possible lumbar puncture.
Aljeshi et al. (22)	20/F/Saudi Arabia	-/2^nd^ dose	Few days	Anxiety; sleep disturbance; nightmares; auditory, visual hallucinations; agitation; aggressive, disorganized behavior; disorientation in time and person; disjointed speech; paranoid delusions.	Unspecified psychosis	8 weeks	No	COVID-19 test: positive.	Olanzapine. Patient was discharged home after complete remission of acute psychotic symptoms with some residual symptoms, mainly reduced concentration and motivation.
Borovina et al. (34)	45/M/Croatia	Viral vector Ad26.COV2.S vaccine/-	5 days	Anxiety; suspiciousness; persecutory delusions; delusions of reference; suicide attempt; low affect modulation.	Unspecified psychosis	4 weeks	No	BT: leukocytes 13.4x109/L, C-RP 158.8 mg/L, ASAT 62 U/L; ALAT 112 U/L; GGT 127 U/L.	Antibiotics; Haloperidol; Alprazolam. After 23 days of treatment in Clinic, the patient was discharged without psychotic symptoms.
	41/M/Croatia	mRNA BNT162b2 vaccine/2^nd^ dose	5 days	Paranoid delusions; severe anxiety; psychomotor agitation; depressed, irritable mood; olfactory hallucinations.	Unspecified psychosis	25 days	No	Within normal limits	Risperidone; Diazepam; Quetiapine; Olanzapine; Fluphenazine; Clonazepam. After 10 days, the patient was discharged with complete remission of his psychotic symptoms.
	35/M/Croatia	mRNA BNT162b2 vaccine/1^st^ dose	2 weeks	Persecutory delusions; delusions of reference; agitation; dysphoric mood.	Unspecified psychosis	1 month	No	Within normal limits	After one month of hospitalization, the patient was discharged with partial remission of his psychotic symptoms.
Shukla et al. (23)	17/F/India	Whole-virion inactivated SARS-CoV-2 antigen vaccine/2^nd^ dose	2 days	Restlessness; irrational fear; suspiciousness; talking to herself; reduced self-care; disinhibited behavior; auditory hallucinations; decreased food intake; insomnia.	Unspecified psychosis	8 weeks	No	Within normal limits	Olanzapine; Clonazepam. On 4-week follow-up, the patient reported that auditory hallucinations had stopped and improvement was noted in symptoms like fearfulness, self-care, and social interaction.
Fekih-Romdhane et al. (24)	26/F/Tunisia	mRNA BNT162b2 vaccine/2^nd^ dose	1 month	Acute psychomotor agitation; incoherent speech; total insomnia; anxiety; delusions of persecution and reference; auditory hallucinations; disorganized speech and behavior.	Lupus cerebritis	1 month	No	BT: severe acute kidney failure, proteinuria, high C-RP values, pancytopenia; strongly positive anti-SSA, anti-Ro52, anti-SSB, weakly positive anti-nucleosome, and anti-histone antibodies. X-ray: bilateral pleural effusion. MRI: hyperintense signals in the left fronto-parietal lobes and the cerebellum.	Haloperidol; Diazepam; Hydroxychloroquine; Prednisone; Cyclophosphamide; Risperidone. During outpatient follow-up, the patient was free from neurological and psychiatric symptoms and had good tolerance to treatment.
Krishna et al. (25)	46/F/India	Viral vector Ad26.COV2.S vaccine/2^nd^ dose	3 days	Muttering to self; insomnia and decreased appetite; irrational fear; auditory hallucinations; reduced self-care; slow psychomotor activity.	Unspecified psychosis	20 days	No	BT: mild leukocytosis.	Risperidone; Clonazepam. The patient came for regular follow-up for continuous 3 months, reported a complete improvement and was back on regular routine.
Neves et al. (26)	38/F/Brazil	mRNA BNT162b2 vaccine/1^st^ dose	1 week	Aggressiveness; bizarre behavior; agitation; lacking self-orientation; delusions of persecution; grandiose delusions; dysphoric mood; restlessness; tachylalia.	Refractory psychosis	6 months	No	Within normal limits	Haloperidol; Valproate; Olanzapine; Clozapine; Risperidone; Lithium carbonate; Aripiprazole. The patient remained hostile, persecutory, and refusing to talk to staff, so her psychosis was defined as refractory, according to IPAP criterion.
Yadav et al. (27)	18/F/India	-/1^st^ dose	Same day	Abnormal paranoid behavior; insomnia; fearfulness; smiling and muttering to self; not responding to verbal commands; negativism, rigidity, mutism and staring.	Guillain barre syndrome	1 week	No	BT: leukocytosis, raised ESR, urea. CSF: elevated protein, glucose and vitamin B12. MR venogram: transverse and sigmoid sinus thrombosis	Olanzapine; Lorazepam; IVIG; LMWH. The patient was moved to an inpatient rehabilitation facility where she spent the next two months undergoing intensive physical, occupational, and supportive therapy.
Chang et al. (28)	39/F/Taiwan	Viral vector ChAdOx1 nCoV-19 vaccine/1^st^ dose	2 days	Delusional parasitosis (patient was very sure to feel 5–7 cm worms crawling under skin and used a blade to cut through the dermis layer to find worms).	Ekbom syndrome	8 months	No	MRI: gliosis over bilateral frontal lobes.	Aripiprazole; Valproic acid. The patient’s mood lability and cognitive deficit were gradually subsided after 12- week of treatment.
		Viral vector ChAdOx1 nCoV-19 vaccine/2^nd^ dose	Few days	Irritable mood; overactivity; spending spree; insomnia; agitation; frequent conflicts; overdose of sleeping pills and beta blockers.	Acute mania with psychotic symptoms				
Al-Mashdali et al. (29)	32/M/Qatar	mRNA-1273 vaccine/1^st^ dose	1 day	Agitation; disorientation to time, place, person; memory disturbances; aggressiveness; auditory hallucinations; bizarre behaviors.	Unspecified psychosis	9 days	No	CSF: elevated protein levels (0.76 gm/L),. EEG: findings suggestive of acute encephalopathy (slowed background activity).	Ceftriaxone; Acyclovir; Haloperidol; Lorazepam; Methylprednisolone. At follow-up in outpatient clinic (one month after the discharge), the patient was asymptomatic and fully oriented.
Simanungkalit et al. (30)	29/F/Indonesia	Whole-virion inactivated SARS-CoV-2 antigen vaccine/2^nd^ dose	1 day	Focal and generalized seizures; auditory hallucinations; fear/paranoia; insomnia; chattering; abnormal movements (dyskinesia, dystonia, opisthotonos); bizarre behaviors.	Anti-NMDAR encephalitis	6 weeks	No	CSF: lymphocytic pleocytosis (43 cells/mm3); positive for anti-NMDAR antibodies. EEG: “delta brush” epileptiform waves of the right frontal lobe.	Methylprednisolone; Immunoglobulins; Rituximab; Antiepileptics and antipsychotics. At follow-up evaluation conducted 3 months after, there was resolution of residual psychotic symptoms.
Kita et al. (31)	37/M/Japan	mRNA-1273 vaccine/3^rd^ dose	4 days	Talkativeness; grandiose delusions; emotional instability; insomnia; excitement; hyperactivity; sexual deviance; hyperthymia; religious delusions.	Acute mania with psychotic features	2 months	No	BT: CRP 0.10 mg/dL; WBC 16,600/µL.	Olanzapine. The patient was discharged from hospital given that his acute mania had completely resolved without administration of any further medication. The patient remained stable and has not exhibited any manic symptoms during follow-up.
Laxmi & Grover (32)	40/F/India	Viral vector ChAdOx1 nCoV-19 vaccine/1st dose	Same day	Insomnia; fearfulness; suspiciousness; delusions of persecution; auditory hallucinations.	Schizophrenia	5 months	No	Within normal limits	Olanzapine; aripiprazole; clozapine. The patient was on regular follow−up and maintaining well on clozapine 75 mg/day. The patient did not receive the second dose of the vaccine.

BT, blood test; ALAT, alanine aminotransferase; ASAT, aspartate aminotransferase; ESR, erythrocyte sedimentation rate; CT, computed tomography; MRI, magnetic resonance imaging; FLAIR, fluid attenuated inversion recovery; C-RP, C-reactive protein; WBC, white blood cells; CPK, creatine phosphokinase; CK-MB, creatine phosphokinase-MB; Hb, hemoglobin; IgG, immunoglobulin G; GGT, gamma-glutamyl transferase; anti-SSA, anti–Sjögren’s-syndrome-related antigen A; anti-SSB, anti-Sjögren’s syndrome antigen B; LMWH, low-molecular-weight heparin; CSF, cerebrospinal fluid; EEG, electroencephalogram; NMDAR, N-methyl-D-aspartate receptor; IVIG, intravenous immunoglobulin.

### 3.2 Patients’ characteristics

Out of the 24 cases, 13 were female (54.2%), and the mean age across all participants was 33.71 ± 12.02 years, with a median of 36 years. A total of 22 (91.2%) patients had no specific history of somatic illness and comorbidities (see **Table 2**).

**Table 2:** The demographic data and medical history of respondents with new-onset psychosis following COVID-19 vaccination.

Variable		N (%)
**Sex**	Male	11 (45.8%)
	Female	13 (54.2%)
**Age**	Mean ± SD	33.71 ± 12.02
	Median	36
	Range	15-57
**Medical history**	Non-syndromic retinitis pigmentosa	1 (4.2%)
	Diabetes mellitus II	1 (4.2%)
	Hypothyroidism	2 (8.3%)
	Hypertension	1 (4.2%)
	Febrile seizures	1 (4.2%)
	No specific medical history	22 (91.2%)

### 3.3 Vaccine characteristics

Administration of the mRNA BNT162b2 vaccine potentially induced adverse psychiatric events in 33.3% of cases, while psychotic symptoms appeared in 25% of cases following the viral vector ChAdOx1 nCoV-19 vaccine. The mean time for the onset of psychotic symptoms was 5.75 ± 8.14 days, which, in most cases, was reported after the first (45.8%) or the second dose (50%), followed by the third dose (4.2%). There was no data on the onset of adverse events in 4.2% of cases (see **Table 3**).

**Table 3:** Characteristics of vaccines and onset of psychotic symptoms following COVID-19 vaccination .

Variable		N (%)
**Vaccine Subtypes**	mRNA BNT162b2 vaccine	8 (33.3%)
	mRNA-1273 vaccine	3 (12.5%)
	Unspecified mRNA vaccine	2 (8.3%)
	Viral vector ChAdOx1-S/nCoV-19 vaccine	6 (25%)
	Viral vector Ad26.COV2.S vaccine	1 (4.2%)
	Whole-virion inactivated BBV152 vaccine	2 (8.3%)
	No data	2 (8.3%)
**Vaccine dose**	1st	11 (45.8%)
	2nd	12 (50%)
	3d	1 (4.2%)
**Onset of psychotic symptoms (days)**	0-2	10 (41.7%)
	3-7	9 (37.5%)
	8-14	2 (8.3%)
	15-28	0
	>28	2 (8.3%)
	No data	1 (4.2%)
	Mean ± SD	5.75 ± 8.14

### 3.4 Clinical characteristics

Almost all reviewed cases (95.8%) presented with psychotic symptoms, such as hallucinations (visual, auditory, olfactory, and tactile) and delusions (mostly persecutory and delusions of reference). The most occurring form of hallucinations was auditory (54.2%), while visual hallucinations were present in 12.5% of cases. Motor disturbances, such as increased or decreased motor activity and bizarre behavior, were mentioned in 83.3% of cases. In 3 (12.5%) cases, a suicidal attempt was described. The largest number of patients presented with unspecified psychosis (54.2%), while mania with psychotic symptoms was detected in 16.7%. The duration of psychotic symptoms was mostly between 1 and 2 months (41.7%) with a mean of 52.48 ± 60.07 days. Details of patients’ clinical presentations are available in **Table 4**.

**Table 4:** Clinical characteristics of new-onset psychosis following COVID-19 vaccination.

Variable		N (%)
**Psychopathological symptoms**	Psychotic symptoms: hallucinations, delusions, paranoia	23 (95.8%)
	Motor disturbances: agitation, increased/decreased motor activity, bizarre movements	20 (83.3%)
	Disturbances of mood: irritability, aggressiveness, anxiety	17 (70.8%)
	Insomnia	12 (50%)
	Disturbances of the consciousness	6 (25%)
	Aphasia	1 (4.2%)
	Suicidal attempt	3 (12.5%)
**Diagnosis**	Unspecified psychosis	13 (54.2%)
	Mania with psychotic symptoms	4 (16.7%)
	Acute & Transient psychotic disorder	1 (4.2%)
	Schizophrenia	1 (4.2%)
	Guillain-Barre syndrome	1 (4.2%)
	Encephalitis	3 (12.5%)
	Lupus cerebritis	1 (4.2%)
**Duration of psychosis**	<2 weeks	5 (20.8%)
	2-4 weeks	5 (20.8%)
	1-2 months	10 (41.7%)
	> 2 months	3 (12.5%)
	No data	1 (4.2%)
	Mean ± SD (days)	52.48 ± 60.07

### 3.5 Laboratory and radiological findings

Results of laboratory and radiological tests were mostly available for blood tests and magnetic resonance imaging (MRI). Abnormalities in blood tests were described in 50% of cases, with the most common being mild to moderate leukocytosis and an elevated C-reactive protein (C-RP) level. MRI results were abnormal in 20.8%, and in most cases, they showed fluid-attenuated inversion recovery hyperintensity in the white matter. In some cases, lumbar puncture with cerebrospinal fluid (CSF) analysis showed elevated protein levels, lymphocytic pleocytosis, positive immunoglobulin G (IgG) oligoclonal bands, and high interleukin (Il)-1 beta. There was one case with a positive COVID-19 test (see **Table 5**).

**Table 5:** Summary of laboratory and radiological findings.

Variable		N (%)
**Blood tests**	Normal	11 (45.8%)
	Abnormal	12 (50%)
	No data	1 (4.2%)
**COVID-19 test**	Positive	1 (4.2%)
	Negative	11 (45.8%)
	No data	12 (50%)
**CT**	Normal	12 (50%)
	Abnormal	1 (4.2%)
	No data	11 (45.8%)
**MRI**	Normal	14 (58.3%)
	Abnormal	5 (20.8%)
	No data	5 (20.8%)
**EEG**	Normal	10 (41.7%)
	Abnormal	2 (8.3%)
	No data	12 (50%)
**USG**	Normal	3 (12.5%)
	Abnormal	0
	No data	21 (87.5%)
**X-ray**	Normal	4 (16.7%)
	Abnormal	2 (8.3%)
	No data	18 (75%)
**CSF analysis**	Normal	3 (12.5%)
	Abnormal	5 (20.8%)
	No data	16 (66.7%)
**Urine analysis**	Normal	7 (29.2%)
	Abnormal	0
	No data	17 (70.8%)

CT, Computed tomography; MRI, Magnetic resonance imaging; EEG, Electroencephalogram; USG, Ultrasonography; CSF, Cerebrospinal fluid.

### 3.6 Treatment and outcome

Almost all (83.3%) patients with psychotic symptoms received atypical antipsychotics for medical treatment, while typical antipsychotics were prescribed in 37.5% of cases, and benzodiazepines in 50% of cases. Additionally, 20.8% of patients received steroids, and 25% were prescribed antiepileptic medications. Overall, 12 patients made a full recovery (50%), while other 50% had residual symptoms such as decreased emotional expressions, low affect, or residual psychotic symptoms (see **Table 6**).

**Table 6:** Therapeutic approach and outcome for patients with new-onset psychosis following COVID-19 vaccination.

Variable		N (%)
**Psychotropic medication**	Typical antipsychotics	9 (37.5%)
	Atypical antipsychotics	20 (83.3%)
	Benzodiazepines	12 (50%)
	Antidepressants	1 (4.2%)
	Mood stabilizers/anticonvulsants	6 (25%)
**Non-psychotropic medication**	Steroids	5 (20.8%)
	Monoclonal antibodies	2 (8.3%)
	Antiviral medications	2 (8.3%)
	Chloroquine derivative	1 (4.2%)
	Antibiotics	3 (12.5%)
	Immune suppressants	1 (4.2%)
	Antihistamine	1 (4.2%)
	DMARDs	1 (4.2%)
	Immunoglobulin	3 (12.5%)
	Anticholinergics, antipyretics, anticoagulants	5 (20.8%)
	No data	1 (4.2%)
**Outcome**	Full recovery	12 (50%)
	Residual symptoms	12 (50%)

DMARDs, Disease-modifying antirheumatic drugs.

### 3.7 Quality assessment

Nineteen case reports and two case series were assessed using the JBI checklist. All case reports (n = 19) had a low risk of bias, while both case series had a moderate risk of bias. Data are shown in **Supplementary Tables 1**, **2**.

## 4 Discussion

The first COVID-19 vaccine was introduced in December 2020, marking the beginning of the fight against the pandemic (35). Currently, available vaccines against SARS-CoV-2 are produced using one of the following technologies: (a) mRNA-based vaccines, (b) viral vector-based vaccines, (c) protein subunit vaccines, and (d) whole virus or inactivated virus vaccines (36–38). More than 13 billion vaccine doses have been administered to date, according to the World Health Organization (1).

Our study of published case reports indicates a marginal disparity in the onset of primary psychosis post-COVID-19 vaccinations between genders. Among the cases reviewed, 54.2% involved females, and 45.8% involved males. Previous cohort studies and reviews have reported an association between the female gender and a higher incidence of side effects following COVID-19 vaccination. These effects encompass typical local and systemic reactions, notably redness, swelling, fever, muscle pain, headache, or anaphylaxis (39, 40). However, a comprehensive systematic review assessing the global incidence of psychotic disorders revealed a cumulative frequency of 26.6 per 100,000 individuals, with men being at a higher risk for all psychotic disorders (41).

Based on the demographic data obtained, the average age of those susceptible to primary psychosis after vaccination is 36 years, with a range of 15 to 57 years. This observation is consistent with results from phase 3 randomized controlled trials and federally sponsored surveillance programs evaluating the safety and efficacy of mRNA COVID-19 vaccines. These studies revealed that both injection-site and systemic adverse events were more prevalent among younger participants aged 18 to less than 65 years (42, 43). Nevertheless, it’s crucial to note the controversial comparison between common side effects following vaccination and rare side effects, such as primary psychosis. Additionally, previous studies indicate that the onset of psychotic symptoms in schizophrenia spectrum disorders commonly occurs between the ages of 15 and 35 years (44). It is crucial to emphasize that cases of post-vaccination psychosis necessitate careful follow-up for the potential development of schizophrenia.

In the majority of cases, applicants lacked a specific medical history pertaining to potential comorbidities. It is worth noting that in one of the described cases, a positive COVID-19 test was obtained. Previous studies have shown that individuals with documented comorbidities and a history of COVID-19 infection exhibit a statistically significant increase in adverse events following vaccination (45, 46).

The average duration of psychosis in the described cases was 52.48 ± 60.07 days. Most authors refrain from specifying a particular diagnosis according to any classification, instead presenting cases of unspecified psychosis characterized by several symptoms, including delusions, hallucinations, bizarre behavior, and insomnia. Although it is challenging to determine the nature of psychosis in these cases, whether it aligns more with organic mental disorders or within schizophrenia spectrum disorders.

Guidelines for the treatment of psychosis and schizophrenia recommend starting treatment with antipsychotic medications, and the choice of antipsychotic drug depends on numerous patient-specific factors (47, 48). According to findings from another study, there appears to be no difference in effectiveness between atypical and typical antipsychotics in the treatment of primary psychosis, but there exists a distinct difference in the side effect profiles (49). Likewise, our study revealed that in the majority of new-onset psychosis cases subsequent to COVID-19 vaccination, atypical antipsychotics (83.3%) were prescribed as the primary pharmacological treatment. In cases of diagnosed or suspected autoimmune encephalitis, steroids were most frequently utilized (20.8%), either as monotherapy or in combination with antipsychotics. It is important to note that, according to the results of our review, 50% of the described cases achieved full recovery, while the second part of the patients retained some residual symptoms. Also, based on the available information, continuation of therapy in the prescribed regimen was necessary in 58.3% of cases, with an average duration of ≥3 months with regular check-ups, which also aligns with clinical guidelines.

Based on the outcomes of our review, the onset of primary psychosis occurred in most cases following vaccination with the mRNA BNT162b2 vaccine (33.3%), closely followed in frequency by cases of new-onset psychosis following vaccination with the viral vector ChAdOx1nCoV-19 vaccine (25%). Previous comparisons have demonstrated that viral vector vaccines tend to be associated with more systemic side effects compared to mRNA vaccines (50). It’s essential to highlight that in the United States and Europe, the mRNA vaccine has been administered in the vast majority of cases (51, 52). However, the results of the clinical study on systemic allergic reactions to COVID-19 vaccination showed that hallucinations were observed in only 0.81% of cases as a psychiatric side effect following the administration of a placebo and subsequent two doses of the mRNA-1273 vaccine (53). This variance in vaccine administration statistics could potentially influence the prevalence of reported side effects, the number of publications, and available data regarding adverse effects associated with each vaccine type.

The majority of patients experienced psychosis following the administration of the first (45.8%) and second (50%) vaccine doses, with noticeable variation in the incidence of primary psychosis after receiving the third dose (4.2%). In general, symptoms manifested rapidly within 0-7 days after vaccination. Enhanced monitoring of psychiatric side effects in the initial weeks following COVID-19 vaccination may be advisable.

According to available data, the occurrence of psychosis following vaccination may be mediated by the body’s immune response to SARS-CoV-2. Specifically, vaccine administration induces a cellular immune response, triggering T-helper cell-mediated release of proinflammatory cytokines. In some instances, this cascade may lead to cytokine storms and hypofunction of N-methyl-D-aspartate (NMDA) receptors. Consequently, elevated dopamine levels may result, potentially precipitating the development of psychosis (17).

Given the presence of increased inflammatory markers in certain psychiatric disorders (54–56), it is plausible to assume that this inflammatory condition could underlie various neuropsychiatric complications associated with vaccination. Likewise, the results of our study revealed elevated C-RP levels and mild to moderate leukocytosis as the most common blood abnormalities. Furthermore, CSF analysis indicated increased protein levels, lymphocytic pleocytosis, and high levels of Il-1 beta, confirming the activation of the inflammatory cascade as well.

Another hypothesis regarding post-vaccination psychosis suggests that the observed alterations in mental status, including psychotic symptoms, could represent a manifestation of autoimmune anti-NMDA encephalitis (15, 57). Cases of diagnosing anti-NMDA encephalitis were also observed in our review. In turn, instances of anti-NMDA encephalitis development have been repeatedly reported following vaccination against other infections, such as yellow fever, influenza, typhus, and pertussis (58–60). Considering the potential link between post-vaccination psychosis and autoimmune anti-NMDA encephalitis, it is advisable to consider immunological screening in individuals presenting psychiatric symptoms post-COVID-19 vaccination.

It is noteworthy that due to various speculations and uncertainties regarding the safety of COVID-19 vaccines, the population is experiencing significant stress, which could also provoke the development of psychiatric reactions (61, 62).

## 5 Limitations

This study has several limitations. First, there are only a few published studies to date on psychiatric side effects following COVID-19 vaccines, resulting in small sample sizes. Second, our study included case reports only in English and only full-text studies, which significantly reduces the sample size. Another limitation is that, since there is no data from a matched control group, morbidity or prevalence cannot be calculated properly. Fourth, limitation is that in our review, there was no assessment of the risk of developing psychosis prior to vaccination in the individuals described in the clinical cases, which is associated with the complexity of conducting this task retrospectively.

## 6 Conclusion

Studies on psychiatric side effects post-COVID-19 vaccination are scarce. Conclusions on vaccine types’ advantages or disadvantages are challenging due to insufficient evidence. Vaccination is generally safe, but data suggest a potential link between young age, mRNA, and viral vector vaccines with new-onset psychosis within 7 days post-vaccination. Collecting data on vaccine-related psychiatric effects is crucial for prevention strategies. An algorithm for monitoring and treating mental health reactions post-vaccination is necessary for comprehensive management of psychiatric complications.

## Data availability statement

The original contributions presented in the study are included in the article/**Supplementary Material**. Further inquiries can be directed to the corresponding author.

## Author contributions

ML: Writing – review & editing, Writing – original draft, Visualization, Software, Resources, Methodology, Investigation, Formal analysis, Data curation, Conceptualization. LR: Writing – review & editing, Writing – original draft, Supervision, Software, Resources, Project administration, Methodology, Investigation, Funding acquisition, Formal analysis, Data curation, Conceptualization. JV: Writing – review & editing, Validation, Supervision, Resources, Project administration, Methodology, Funding acquisition, Formal analysis, Data curation, Conceptualization. ER: Writing – review & editing, Validation, Supervision, Resources, Project administration, Methodology, Funding acquisition, Formal analysis, Data curation, Conceptualization.
