# Supplementary material for: New-onset psychosis following COVID-19 vaccination: a systematic review
Source: Front Psychiatry. 2024 Apr 12;15:1360338. doi: 10.3389/fpsyt.2024.1360338 (PMC11046000; doi:10.3389/fpsyt.2024.1360338)
Supplement: Supplementary file 1 [file DataSheet_1.pdf]

Supplementary Table 1. JBI Critical Appraisal Checklist for Case Reports

Reference	Q1	Q2	Q3	Q4	Q5	Q6	Q7	Q8	Total (%)	ROB
Flannery et al., 2021	No	Yes	Yes	Yes	Yes	Yes	Yes	Yes	7 (87.5%)	Low risk
Reinfeld et al., 2021	Yes	Yes	Yes	Yes	Yes	Yes	Yes	Yes	8 (100%)	Low risk
Grover et al., 2022	No	Yes	Yes	Yes	Yes	Yes	Yes	Yes	7 (87.5%)	Low risk
Lien et al., 2022	No	Yes	Yes	Yes	Yes	Yes	Yes	Yes	7 (87.5%)	Low risk
Takata et al., 2021	Yes	Yes	Yes	Yes	Yes	Yes	Yes	Yes	8 (100%)	Low risk
Roberts et al., 2021	Yes	Yes	Yes	Yes	Yes	Yes	Yes	Yes	8 (100%)	Low risk
Renemane et al., 2022	Yes	Yes	Yes	Yes	Yes	Yes	Yes	Yes	8 (100%)	Low risk
Alphonso et al., 2022	Yes	Yes	Yes	Yes	Yes	Yes	Yes	Yes	8 (100%)	Low risk
Aljeshi et al., 2022	No	Yes	Yes	Yes	No	Yes	Yes	Yes	6 (75%)	Low risk
Shukla et al., 2023	No	Yes	Yes	Yes	Yes	Yes	Yes	Yes	7 (87.5%)	Low risk
Fekih-Romdhane et al., 2023	Yes	Yes	Yes	Yes	Yes	Yes	Yes	Yes	8 (100%)	Low risk
Krishna et al., 2022	Yes	Yes	Yes	Yes	Yes	Yes	Yes	Yes	8 (100%)	Low risk
Neves et al., 2023	No	Yes	Yes	Yes	Yes	Yes	Yes	Yes	7 (87.5%)	Low risk
Yadav et al., 2023	Yes	Yes	Yes	Yes	No	Yes	Yes	Yes	7 (87.5%)	Low risk
Chang et al., 2023	Yes	Yes	Yes	Yes	Yes	Yes	Yes	Yes	8 (100%)	Low risk
Al-Mashdali et al., 2021	Yes	Yes	Yes	Yes	Yes	Yes	Yes	Yes	8 (100%)	Low risk
Simanungkalit et al., 2022	No	Yes	Yes	Yes	Yes	Yes	Yes	Yes	7 (87.5%)	Low risk
Kita et al., 2023	Yes	Yes	Yes	Yes	Yes	Yes	Yes	Yes	8 (100%)	Low risk
Laxmi & Grover, 2023	Yes	Yes	Yes	Yes	Yes	Yes	Yes	Yes	8 (100%)	Low risk
**Abbreviations**: ROB, Risk of bias										

Supplementary Table 2. JBI Critical Appraisal Checklist for Case Series

Reference	Q1	Q2	Q3	Q4	Q5	Q6	Q7	Q8	Q9	Q10	Total (%)	ROB
Yesilkaya et al., 2021	Yes	Yes	Yes	UC	UC	Yes	Yes	Yes	No	NA	6 (60%)	Moderate risk
Borovina et al., 2022	Yes	Yes	Yes	UC	UC	No	Yes	Yes	No	NA	5 (50%)	Moderate risk

Abbreviations:
NA, not applicable; ROB, Risk of bias; UC, unclear.
